# Transcriptomic and genetic approaches reveal an essential role of the NAC transcription factor SlNAP1 in the growth and defense response of tomato

**DOI:** 10.1038/s41438-020-00442-6

**Published:** 2020-12-25

**Authors:** Jiao Wang, Chenfei Zheng, Xiangqi Shao, Zhangjian Hu, Jianxin Li, Ping Wang, Anran Wang, Jingquan Yu, Kai Shi

**Affiliations:** Department of Horticulture, Zhejiang University, 866 Yuhangtang Road, 310058 Hangzhou, People’s Republic of China

**Keywords:** Biotic, Molecular engineering in plants

## Abstract

With global climate change, plants are frequently being exposed to various stresses, such as pathogen attack, drought, and extreme temperatures. Transcription factors (TFs) play crucial roles in numerous plant biological processes; however, the functions of many tomato (*Solanum lycopersicum* L.) TFs that regulate plant responses to multiple stresses are largely unknown. Here, using an RNA-seq approach, we identified *SlNAP1*, a NAC TF-encoding gene, which was strongly induced by various stresses. By generating *SlNAP1* transgenic lines and evaluating their responses to biotic and abiotic stresses in tomato, we found that *SlNAP1*-overexpressing plants showed significantly enhanced defense against two widespread bacterial diseases, leaf speck disease, caused by *Pseudomonas syringae* pv. *tomato* (*Pst*) DC3000, and root-borne bacterial wilt disease, caused by *Ralstonia solanacearum*. In addition, *SlNAP1* overexpression dramatically improved drought tolerance in tomato. Although the *SlNAP1*-overexpressing plants were shorter than the wild-type plants during the early vegetative stage, eventually, their fruit yield increased by 10.7%. Analysis of different hormone contents revealed a reduced level of physiologically active gibberellins (GAs) and an increased level of salicylic acid (SA) and abscisic acid (ABA) in the *SlNAP1*-overexpressing plants. Moreover, EMSAs and ChIP-qPCR assays showed that SlNAP1 directly activated the transcription of multiple genes involved in GA deactivation and both SA and ABA biosynthesis. Our findings reveal that SlNAP1 is a positive regulator of the tomato defense response against multiple stresses and thus may be a potential breeding target for improving crop yield and stress resistance.

## Introduction

With global climate change, various stresses, such as pathogen attack, drought, and extreme temperatures, are occurring more frequently across the world. As sessile organisms, plants are faced with such biotic and abiotic stresses in situ, which often drastically affect their growth and cause tremendous economic losses. Plants have evolved sophisticated defense mechanisms to precisely sense and respond to pathogen attack and abiotic stresses. Briefly, plant cells detect environmental stimuli via specific sensors or receptors, which trigger downstream responses. These responses occur via the reactive oxygen species (ROS) burst; phosphorylation of mitogen-activated protein (MAP) kinases; transcriptional reprogramming; and metabolic changes at physiological, biochemical, cellular and molecular levels.

Transcription factors (TFs) play crucial roles in the transcriptional reprogramming of stress-related genes^1–3^. In particular, NAC proteins such as NAM, ATAF1/2, and CUC2 compose a large plant-specific TF family and have a highly conserved N-terminal domain that functions as a DNA-binding domain (known as the NAC domain) and a variable C-terminal domain that is responsible for transcriptional regulation. Extensive studies have revealed that NAC TFs are involved in a wide variety of plant biological processes, including plant growth and development, leaf senescence, secondary wall formation, and responses to biotic and abiotic stresses^4–11^. Nonetheless, the functions of NAC TFs are highly species specific. For instance, transgenic plants overexpressing *ATAF1* show reduced resistance to *Pseudomonas syringae* pv. *tomato* (*Pst*) DC3000, *Botrytis cinerea* (*B. cinerea*) and *Alternaria brassicicola* in Arabidopsis^12,13^, while the ATAF1 homolog in rice, OsNAC6, plays a positive role in plant defense, as transgenic plants overexpressing *OsNAC6* show enhanced resistance to *Magnaporthe grisea*^14^. Similarly, two closely related NAC TFs in tomato, jasmonic acid 2 (JA2) and JA2-like (JA2L), play opposite roles in immunity against *Pst* DC3000 by differentially regulating stomatal closure and reopening^15^. *Solanum lycopersicum* stress-related NAC1 (SlSRN1) functions positively in the defense against *Pst* DC3000 and *B. cinerea*, while SlSRN1 acts as a negative regulator of drought tolerance in tomato^16^. Thus, the functions of NAC proteins in response to different stresses are complex and still obscure.

Many previous studies have demonstrated that some NAC TFs, such as SlNAC35 and SlSRN1, induce plant defense responses via salicylic acid (SA) or jasmonic acid (JA) (and their crosstalk) signaling pathways during pathogen attack. Under abiotic stress conditions, SlNAC5, SlNAM1, and other NAC TFs activate the transcription of defense-related genes mainly via the JA or abscisic acid (ABA) signaling pathways^17,18^. It therefore seems that plant hormones play critical roles in the functions of NAC TFs, which is worthy of further investigation.

Tomato (*S. lycopersicum* L.) is one of the most popular vegetable species in the world. However, many tomato cultivars are susceptible to pathogen infections and abiotic stresses, which cause billions of dollars of crop yield losses every year^19,20^. In particular, *Pst* DC3000, which is an aboveground bacterial pathogen that causes leaf speck disease, resulting in a large yield penalty of vegetable crops, has been used as a model pathogen for understanding plant–bacterial interactions since the early 1990s^21^. *Ralstonia solanacearum*, a belowground bacterial pathogen that causes root-borne bacterial wilt disease, is one of the most aggressive and destructive pathogens worldwide due to the high mortality rates of diseased plants and the lack of effective control measures^22^. Moreover, drought is considered one of the most significant abiotic stresses, severely limiting plant growth and crop productivity^9^. There are 101 NAC TFs in tomato, but only ~10–20 NAC TFs have been functionally characterized^18^. Here, using transcriptomic and genetic approaches, we identified a NAC TF, *SlNAP1*, and characterized its functions in defense against *Pst* DC3000 and *R. solanacearum*, as well as in drought tolerance. This study highlights the positive role of SlNAP1 TFs in the defense against multiple stresses in tomato, suggesting that SlNAP1 can be a compelling biotechnological target to improve crop resilience.

## Results

### Changes in the transcript levels of tomato NAC genes in response to *Pst* DC3000 infection

**Fig. 1:**
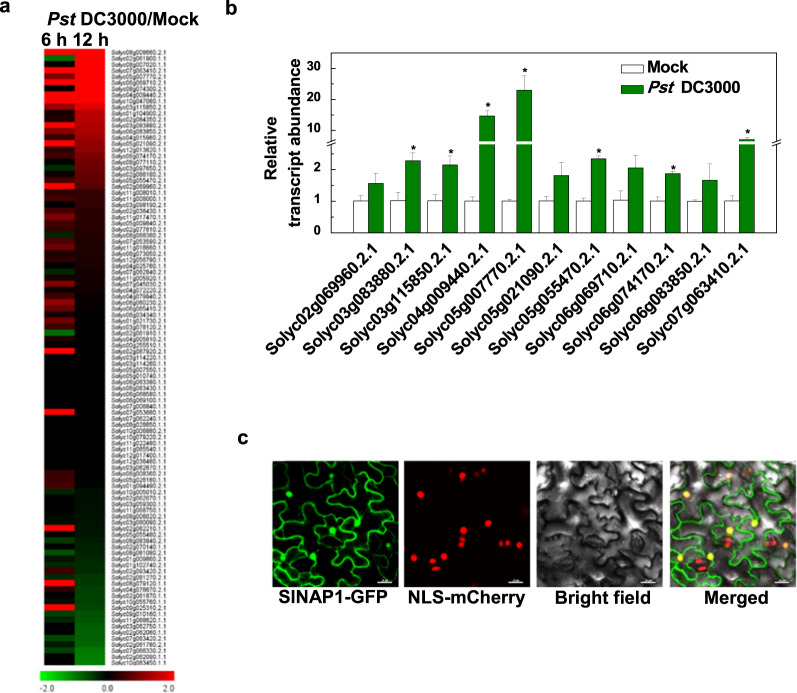
*SlNAP1* expression is strongly induced by *Pst* DC3000 infection. **a** Heatmap showing the fold change (FCh-1) of 101 tomato NAC genes after 6 h and 12 h of *Pst* DC3000 infection. Green, downregulated; red, upregulated (as presented by the color bar). **b** Effects of *Pst* DC3000 infection on the relative transcript abundance of 11 NAC genes in tomato. The leaves were sampled at 0.5 dpi. The transcript abundance of each gene that underwent mock treatment was defined as 1. An asterisk indicates a significant difference between mock- and *Pst* DC3000-inoculated plants at the 5% level of significance (*P* < 0.05). *n* = 3. **c** Subcellular localization of SlNAP1. The tomato SlNAP1-GFP plasmid was transiently expressed in *N. benthamiana* leaves. The GFP and mCherry (a marker for nuclear localization) signals were visualized using confocal microscopy at 48 h after infiltration. Bar = 25 µm

To analyze the expression of tomato NAC genes in response to *Pst* DC3000 infection, we acquired the expression ratio (*Pst* DC3000/mock) data from two transcriptome profiles generated at 6 and 12 h post-inoculation (hpi) with *Pst* DC3000 in tomato leaves. We screened and obtained a list of 101 NAC TFs and their gene expression ratios after *Pst* DC3000 infection (Fig. 1a and Supplementary Table S2). Of these, 11 NAC genes were significantly upregulated by *Pst* DC3000 infection (expression ratio >1.5, and RPKM > 0) (Supplementary Table S2). We further analyzed the expression of these 11 NAC genes at 12 hpi via qRT-PCR. As shown in Fig. 1b, the expression of Solyc05g007770.2.1 (hereafter, SlNAP1) was the greatest after 12 h of *Pst* DC3000 infection, which was more than 23-fold that in the control plants.

To determine the subcellular localization of tomato SlNAP1, a 35S:SlNAP1-GFP construct was transiently expressed in *Nicotiana benthamiana* (*N. benthamiana*) leaves. We found that SlNAP1-GFP localized to the plasma membrane and nucleus (Fig. 1c).

### SlNAP1 inhibits vegetative growth but increases fruit yield of tomato plants

**Fig. 2:**
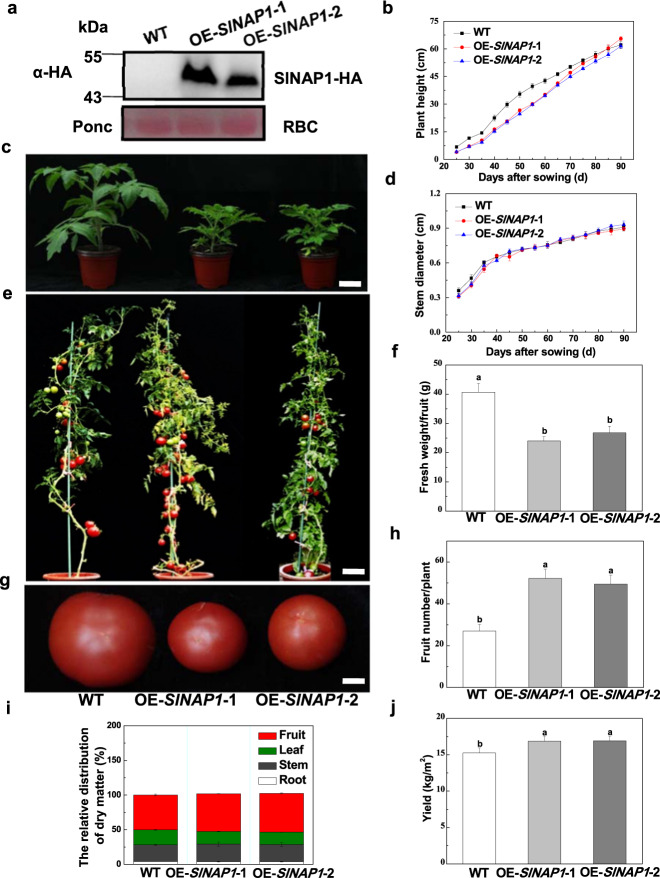
Effects of *SlNAP1* overexpression on plant growth and fruit phenotype. **a** Detection of the SlNAP1-HA fusion protein in two independent homozygous *SlNAP1*-overexpressing lines (OE-*SlNAP1*-1 and -2) by western blot analysis using an anti-HA (Pierce) monoclonal antibody and the large subunit of Rubisco (RBC; as a loading control) in this experiment. **b**, **c**, **d** Plant phenotype in the early growth stage and growth indexes. The images were taken at 40 days (**c**) after sowing. Bar = 5 cm. Plant height and width were determined every 5 days after 25 days of sowing. **e** Plant phenotype in the advanced reproductive stage. Bar = 10 cm. **f**, **g** Fresh weight per fruit (**g**) and ripened fruit phenotype. Bar = 1 cm. **h**, **j** Fruit number per plant and yield (kg/m^2^). **i** Dry matter contents of tomato plant roots, stems, leaves, and fruits. The results are presented as the averages ± SDs of five plants, which were grown in a greenhouse. The different letters indicate significant differences between treatments (*P* < 0.05, Tukey’s test)

To explore the function of SlNAP1 in tomato, we generated *SlNAP1*-overexpressing plants. The SlNAP1-HA fusion protein was detected in two independent homozygous F2 progeny lines, OE-*SlNAP1*-1 and OE-*SlNAP1*-2, by western blot analysis (Fig. 2a). The *SlNAP1*-overexpressing plants presented reduced plant height and leaf area compared with those of the WT plants up to 75 days after sowing (Fig. 2b, c), without a significant difference in their stem diameters (Fig. 2d). The net photosynthesis rate (Pn) of leaves significantly decreased in the *SlNAP1*-overexpressing plants compared with the WT plants (Supplementary Fig. S1). *SlNAP1* overexpression initially inhibited vegetative growth, although the height of *SlNAP1*-overexpressing plants was close to that of the WT plants at 75 days after sowing (Fig. 2b).

Compared to those of the WT plants, the size of the fruits of both transgenic *SlNAP1*-overexpressing plants was smaller (Fig. 2e, g), and the fresh weight per fruit of the OE-*SlNAP1*-1 and OE-*SlNAP1*-2 plants decreased by 41.0% and 34.1%, respectively (Fig. 2f). However, compared with those of the WT plants, the fruit number per plant and the fruit yield of the *SlNAP1*-overexpressing plants increased significantly. The fruit number per plant of the OE-*SlNAP1*-1 and OE-*SlNAP1*-2 plants increased by 92.6% and 81.5%, respectively (Fig. 2h), and the fruit yield increased by 10.7% and 11.0%, respectively (Fig. 2j). The dry matter contents of the roots and stems were nearly the same, whereas the dry matter contents of the leaves and fruits showed significant differences between the *SlNAP1*-overexpressing plants and WT plants (Fig. 2i). Compared to those of the WT plants, the dry matter contents of leaves of the OE-*SlNAP1*-1 and OE-*SlNAP1*-2 plants decreased by 12.9% and 17.1%, respectively, while the dry matter contents of the fruits increased by 11.6% and 14.1%, respectively (Fig. 2i).

### SlNAP1 positively regulates disease resistance and drought tolerance

**Fig. 3:**
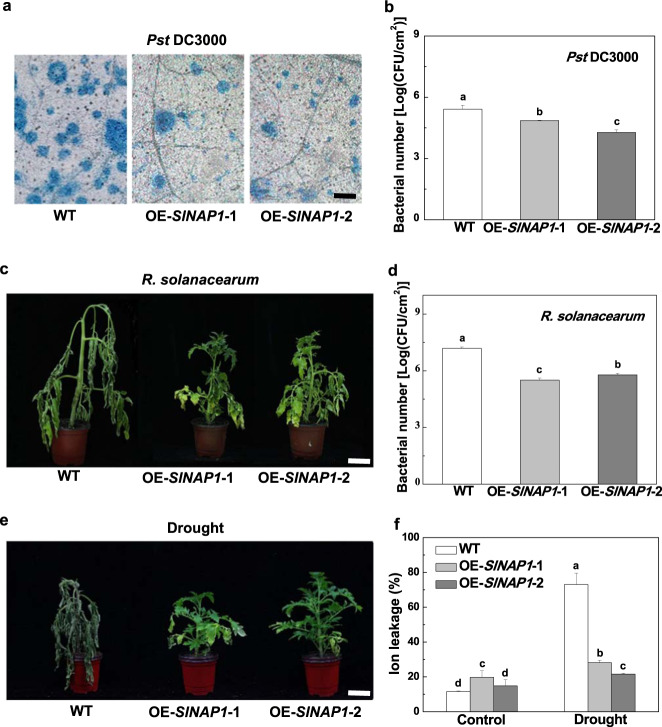
Effects of *SlNAP1* overexpression on tomato disease resistance and drought tolerance. **a** Trypan blue staining for cell death in tomato leaves sampled at 2 dpi with *Pst* DC3000. Bar = 500 µm. **b** *Pst* DC3000 bacterial population at 2 dpi. **c** Phenotypes of plants at 12 dpi with *R. solanacearum*. Bar = 5 cm. **d** *R. solanacearum* bacterial population at 12 dpi. **e** Phenotypes of plants after the imposition of drought for 7 days. Bar = 5 cm. **f** Ion leakage of plant leaves under drought for 7 days. The results represent the averages ± SDs, *n* = 3. The different letters indicate significant differences between treatments (*P* < 0.05, Tukey’s test). The experiments described above were performed three times, each yielding similar results

To determine the role of SlNAP1 in the defense against multiple stresses, first, WT and *SlNAP1*-overexpressing plants were inoculated with *Pst* DC3000. We found that the *SlNAP1*-overexpressing plants showed significantly enhanced resistance to *Pst* DC3000, as reflected by reduced cell death and bacterial populations in tomato leaves compared to those in the WT plants (Fig. 3a, b). Moreover, we inoculated the WT and *SlNAP1*-overexpressing plants with *R. solanacearum*. In this case, the *SlNAP1*-overexpressing plants also showed increased resistance to *R. solanacearum*, as evidenced by a relatively healthy appearance and reduced bacterial populations (Fig. 3c–d). At 12 dpi, the WT plants showed severe wilting, while the *SlNAP1*-overexpressing plants exhibited minor symptoms (Fig. 3c). Taken together, these results indicate that, in tomato, SlNAP1 plays a positive role in the defense against both above- and belowground bacterial pathogens.

Furthermore, the WT and *SlNAP1*-overexpressing plants were subjected to water deprivation for 14 days. Compared with the WT plants, the *SlNAP1*-overexpressing plants showed enhanced tolerance to drought, as reflected by relatively minor wilting and significantly reduced ion leakage in the leaves under drought (Fig. 3e–f). These results demonstrate that SlNAP1 also functions as a positive regulator of drought tolerance in tomato.

### SlNAP1 suppresses the accumulation of GAs and promotes the biosynthesis of SA and ABA

**Fig. 4:**
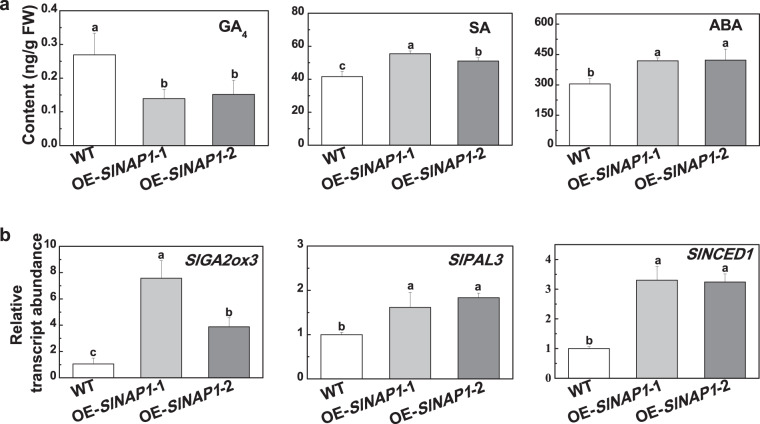
Effects of *SlNAP1* overexpression on endogenous leaf hormone contents and the transcript abundance of hormone metabolism-related marker genes in tomato. **a** Effects of *SlNAP1* overexpression on endogenous hormone contents of tomato leaves. Leaf samples of four-week-old tomato plants were randomly collected from lateral leaflets from the uppermost 1 to 2 fully expanded leaves for the analysis of different hormones. GA_4_ an active gibberellin, SA salicylic acid, ABA abscisic acid. **b** Effects of overexpressing *SlNAP1* on the transcript abundance of the hormone metabolism-related marker genes *SlGA2ox3*, *GIBBERELLIN 2-OXIDASE 3*, which encodes a major GA deactivation enzyme, *SlPAL3*, *PHENYLALANINE AMMONIA-LYASE 3*, which encodes a major SA biosynthesis-related enzyme, *SlNCED1*, *9-CIS-EPOXYCAROTENOID DIOXYGENASE 1*, which encodes a rate-limiting enzyme involved in ABA biosynthesis. The results are shown as the averages ± SDs, *n* = 3. The different letters indicate significant differences (*P* < 0.05, Tukey’s test)

Plant hormones play critical roles in the regulation of plant growth and defense responses to biotic and abiotic stresses. To determine whether SlNAP1 participates in the regulation of plant hormone homeostasis and subsequent plant growth and defense, we first measured the contents of growth- and stress-related plant hormones, such as GA, SA and ABA, in WT and *SlNAP1*-overexpressing plants. As shown in Fig. 4a, the levels of GA_4_, an important biologically active GA in plants, were lower in the *SlNAP1*-overexpressing plants than in the WT plants, while the contents of SA and ABA were higher. Moreover, we analyzed the expression of genes involved in GA, SA and ABA metabolism, including *GIBBERELLIN 2-OXIDASE 3* (*SlGA2ox3*), which encodes a major GA deactivation enzyme, *PHENYLALANINE AMMONIA-LYASE 3* (*SlPAL3*), which encodes a major SA biosynthesis-related enzyme, and *9-CIS-EPOXYCAROTENOID DIOXYGENASE 1* (*SlNCED1*), which encodes a rate-limiting enzyme involved in ABA biosynthesis. The transcript levels of *SlGA2ox3*, *SlPAL3* and *SlNCED1* all significantly increased in the *SlNAP1*-overexpressing plants compared with the WT plants (Fig. 4b). These results imply that SlNAP1 suppresses GA accumulation and promotes SA and ABA biosynthesis by regulating the transcription of *SlGA2ox3*, *SlPAL3* and *SlNCED1* in tomato.

### SlNAP1 directly targets *SlGA2ox3*, *SlPAL3*, and *SlNCED1*

**Fig. 5:**
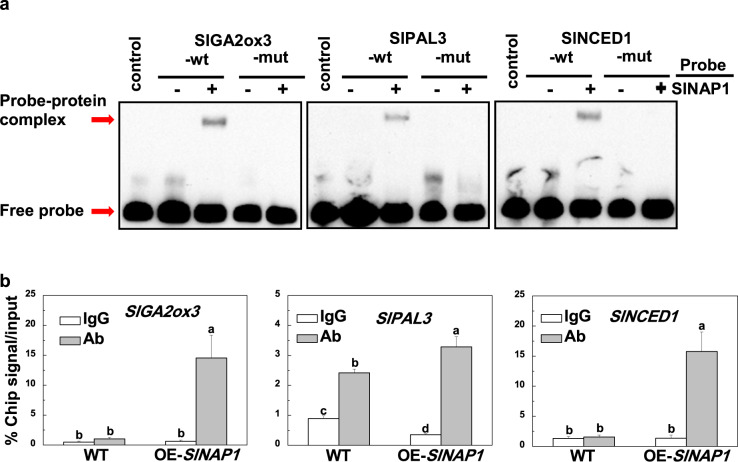
SlNAP1 directly targets *SlGA2ox3*, *SlPAL3*, and *SlNCED1*. **a** Electrophoretic mobility shift assay. A SlNAP1-His fusion protein was incubated with biotin-labeled wild-type (SlGA2ox3-wt) or mutant (SlGA2ox3-mut) SlGA2ox3 oligos, wild-type (SlPAL3-wt) or mutant (SlPAL3-mut) SlPAL3 oligos and wild-type (SlNCED1-wt) or mutant (SlNCED1-mut) SlNCED1 oligos. Proteins purified from the empty vector (EV) were used as negative controls in this experiment. **b** Chromatin immunoprecipitation assay. Samples from wild-type and 35S:SlNAP1-HA tomato plants were precipitated using anti-HA antibodies. A control reaction with goat anti-mouse IgG was simultaneously processed. The ChIP results are shown as percentages of the input DNA. The results are shown as averages ± SDs, *n* = 3. The different letters indicate significant differences (*P* < 0.05, Tukey’s test)

To further confirm whether SlNAP1, a NAC TF, can regulate *SlGA2ox3*, *SlPAL3*, and *SlNCED1* transcription through direct interaction between SlNAP1 and the promoter regions of target genes, we conducted an EMSA in vitro. Typically, NAC TFs preferentially bind to CACG, which is referred to as the NAC core-binding site, of their target promoters^23^. Sequence analysis showed that the promoters of *SlGA2ox3*, *SlPAL3*, and *SlNCED1* all had a NAC core-binding site. We then designed DNA probes that encompassed the NAC core-binding site and generated SlNAP1-His fusion proteins. The results showed that the SlNAP1-His fusion protein could bind to the promoters of *SlGA2ox3*, *SlPAL3* and *SlNCED1* and cause a mobility shift (Fig. 5a). When the NAC core binding sequences in the *SlGA2ox3*, *SlPAL3*, and *SlNCED1* probes were mutated (-mut), the binding ability of SlNAP1 to the probes was lost (Fig. 5a). We then conducted ChIP assays to determine the direct interaction between SlNAP1 and the promoters of the three genes in vivo. After ChIP-qPCR analysis with an anti-HA antibody, the *SlGA2ox3*, *SlPAL3*, and *SlNCED1* promoters were significantly enriched in the OE-SlNAP1-HA samples compared with those in the WT control sample, while the IgG control was not enriched (Fig. 5b). These results suggest that SlNAP1 could directly target *SlGA2ox3*, *SlPAL3*, and *SlNCED1* to regulate the metabolism of relevant hormones in tomato plants.

## Discussion

Extreme weather events are occurring more frequently with global climate change. Thus, plants are faced with more adverse environmental conditions imposed by multiple stresses at the same time. TF-based genetic engineering is a powerful tool improving crop tolerance to stress. However, very few TFs that can regulate plant responses to multiple stresses have been functionally characterized in tomato. In this study, we screened *SlNAP1* from among all the members of the whole tomato NAC TF family (Fig. 1) and found that SlNAP1 can significantly enhance defense against multiple stresses and increase fruit yields (Figs. 2 and 3). In-depth molecular investigations revealed that SlNAP1 directly activated the transcription of genes involved in GA deactivation, as well as SA and ABA biosynthesis (Figs. 4 and 5). Our findings suggest that the tomato NAC TF SlNAP1 is a potentially valuable candidate for genetic engineering programs aiming to generate high-yielding crops that are tolerant to multiple stresses.

NAC TFs have been shown to play dual roles (positive and negative) in the plant defense response, depending on the stresses and plant species. Here, we found that SlNAP1 can significantly increase defense against multiple stresses, including defense against both above- and belowground bacterial pathogens, namely, *Pst* DC3000 and *R. solanacearum*, and tolerance to drought (Fig. 3). Notably, the tomato SlNAP2 and Arabidopsis AtNAP (Arabidopsis ortholog of SlNAP1) SlNAP1 homologs have been identified as central positive regulators of leaf senescence^6,7^. There are several other functionally identified NAC TFs that are involved in the defense response to stresses in tomato. For example, JA2 and JA2L regulate immunity against bacterial pathogens^15^. Another NAC TF, *S. lycopersicum* JUNGBRUNNEN 1 (SlJUB1), plays a positive role in tomato tolerance to drought^9^. Based on these studies, it appears that these NAC TFs regulate only a single stress response in tomato, while SlNAP1 regulates the response to multiple stresses. Additionally, we found that SlNAP1 inhibited the growth but increased the fruit yield of tomato plants. Compared with the WT plants, *SlNAP1*-overexpressing plants were shorter and had concomitantly smaller leaves (Fig. 2b–c), the phenotype of which was similar to that of transgenic tomato lines overexpressing *AtJUB1*^9^. SlNAP1 significantly increased the fruit number per plant and fruit yield, possibly by allocating more photoassimilates from the leaves to the fruits (Fig. 2h–j), implying that there may be a trade-off between vegetative and reproductive development. Similarly, life history theory indicates that the vegetative and reproductive functions of plants compete for common limited resources. An increased carbon allocation to reproductive development results in a relative reduction in vegetative growth^24,25^. Overall, SlNAP1 seems to be an essential NAC TF that plays critical roles in the defense against multiple stresses and the regulation of plant growth and fruit productivity.

During plant-pathogen interactions, SA is recognized as a major plant defense hormone against biotrophic and hemibiotrophic pathogen infection^26^. We found that SlNAP1 played a positive role in the defense against bacterial pathogens by increasing the SA contents and by selectively activating the expression of *SlPAL3*, which is involved in SA biosynthesis (Figs. 4 and 5). Compared with the WT plants, the *SlNAP1*-overexpressing plants had higher SA contents and *SlPAL3* transcript levels (Fig. 4a–b), and the SlNAP1 protein could bind to the promoter regions of *SlPAL3* (Fig. 5a–b). Similarly, a previous study revealed that ectopic overexpression of *SlNAC35* in tobacco increased resistance to *R. solanacearum* by upregulating the expression of the SA-responsive defense genes *PR1a*, *NPR1*, *PR2,* and *PR5*^27^. Conversely, JA2 and JA2L play opposite roles in the defense against bacterial pathogens by regulating pathogen-induced stomatal closure and reopening, respectively^15^. Nonetheless, these NAC TFs are involved in the defense against pathogens via different mechanisms, which may be related to their localization. JA2 and JA2L are expressed predominantly in guard cells^15^; however, SlNAP1 was localized to the mesophyll cells of tomato leaves (Fig. 1c).

In addition, SlNAP1 is involved in drought tolerance. Previous studies demonstrated that a reduced GA level promoted plant tolerance to drought, which was based on the fact that tomato plants overexpressing *AtGAMT1* (which encodes an enzyme that can catalyze the methylation of active GAs to generate inactive GA methyl esters) exhibited typical GA-deficiency phenotypes and increased tolerance to drought^28^. The plant hormone ABA plays a crucial role in the defense against drought stress^1^. Typically, plants synthesize ABA in different tissues to initiate defense responses under stress conditions^29^. Therefore, we analyzed the contents of GAs and ABA in both *SlNAP1*-overexpressing and WT plants. We found that the active GA_4_ content was reduced in the *SlNAP1*-overexpressing plants compared to the WT plants, while the ABA content increased (Fig. 4a). The transcription of *SlGA2ox3* and *SlNCED1* was significantly induced by *SlNAP1* overexpression (Fig. 4b), and the SlNAP1 protein could bind to the promoter regions of *SlGA2ox3* and *SlNCED1* (Fig. 5a–b). These results imply that, as a positive regulator, SlNAP1 could induce drought tolerance, possibly by activating the expression of *SlGA2ox3* and *SlNCED1*, which are involved in GA deactivation and ABA biosynthesis in tomato. Similar to SlNAP1, SlJUB1 also plays a positive role in drought tolerance. SlJUB1 targets *SlDREB1*, *SlDREB2*, and *SlDELLA* to transcriptionally regulate GA signaling pathways^9^. In addition, GAs play a critical role in the regulation of plant growth and act as negative regulators of growth cessation^30^. Transgenic tomato lines overexpressing *AtJUB1* show a stunted phenotype due to GA and BR deficiencies^9^. Thus, we speculate that SlNAP1 directly activates *SlGA2ox3* transcription to inhibit plant growth via GA deactivation in tomato.

Additionally, emerging evidence has indicated the presence of complex crosstalk between different hormones to coordinate plant growth and development, as well as responses to stress. For example, many constitutive defense mutants with elevated SA levels exhibit dwarf plant phenotypes, implying that SA may function antagonistically to GA to modulate plant growth^31^. In contrast, GA synergistically functions with SA in regulating disease resistance^32^. The crosstalk between SA and ABA is mainly antagonistic, and many studies show that ABA can attenuate plant defense responses^33^. Moreover, GA and ABA play antagonistic roles in the regulation of seed germination, root growth, fruit set and responses to abiotic stresses^34,35^. In our study, we found that there were significant differences in the contents of GA, SA and ABA hormones together with altered plant morphology, fruit size, and stress tolerance between the WT and *SlNAP1*-overexpressing plants (Figs. 2–4). We speculate that crosstalk among the three hormones, rather than the activity of a single hormone, might regulate these various processes in *SlNAP1*-overexpressing plants, which needs further investigation. Moreover, SlNAP1 directly activated the transcription of genes involved in the metabolism of these multiple stress-related hormones (Figs. 4 and 5), which appears to be a possible unique feature of SlNAP1 as a positive regulator of both biotic and abiotic stress responses.

**Fig. 6:**
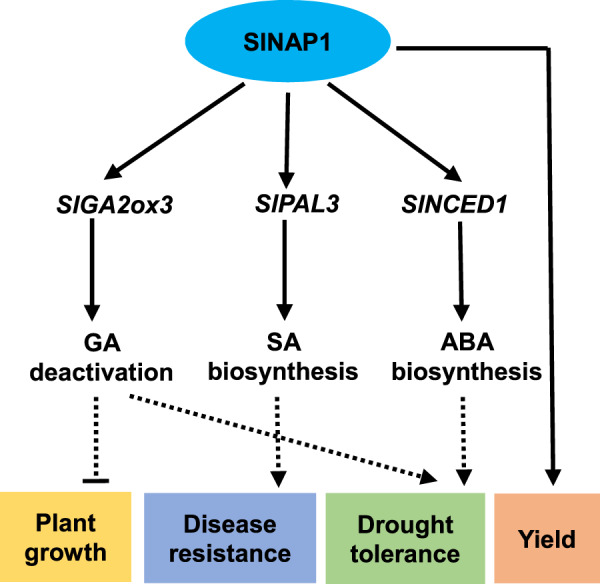
Proposed model for SlNAP1 action in the regulation of plant growth and defense in tomato. SlNAP1 directly activates the transcription of *SlGA2ox3*, *SlPAL3*, and *SlNCED1*, which are involved in GA deactivation, as well as SA and ABA biosynthesis, leading to reduced plant growth and increased disease resistance, drought tolerance, and yield

In conclusion, we identified a vital tomato NAC TF, SlNAP1, which positively regulates the defense against multiple stresses by promoting GA deactivation and by stimulating SA and ABA biosynthesis (Fig. 6), further indicating the importance of NAC TFs in crop improvement.

## Materials and methods

### Plant materials and growth conditions

All the tomato (*S. lycopersicum* L.) lines generated in the present study were in the Condine Red (CR) wild-type (WT) background. To construct transgenic plants overexpressing *SlNAP1*, the coding DNA sequence (CDS) of *SlNAP1* was obtained from tomato cDNA via PCR using specific primers (Supplementary Table S1). The PCR product was subsequently inserted into a pFGC1008-3HA binary plasmid vector under the control of the 35S CaMV promoter. The resulting OE-SlNAP1-3HA plasmid was transformed into tomato (cultivar Condine Red), mediated by *Agrobacterium tumefaciens* strain GV3101. The transgenic plants were further verified by western blotting using anti-HA (Pierce) monoclonal antibodies (Fig. 2a). Two independent F2 progeny homozygous lines (OE-*SlNAP1*-1 and -2) were used for phenotypic and molecular characterization.

Tomato seeds were germinated in 72-well trays in growth medium consisting of a mixture of peat and vermiculite (3:1, v/v). The tomato seedlings were transferred to plastic pots when two true leaves had fully expanded. The growth conditions were as follows: 500 µmol m^−2^ s^−1^ of photosynthetic photon flux density (PPFD), a 13/11 hours (h) (day/night) photoperiod, a 25/21 °C (day/night) air temperature and 80% relative humidity. Approximately 4 weeks after sowing, tomato seedlings at the ~5-leaf stage were used for each treatment.

### Pathogen inoculation and disease symptom assays

The bacterium *Pst* DC3000 was cultured in King’s B solid media consisting of 25 mg mL^−1^ rifampicin at 28 °C overnight and resuspended in 10 mM MgCl_2_. Whole plants were inoculated with the bacterial suspension at a concentration of 10^7^ colony-forming units (CFU) mL^−1^ together with 0.02% Silwet L-77 by spraying^36^. Trypan blue staining and bacterial population counting (CFU) were conducted at 2 days post-inoculation (dpi) to assess disease symptoms^37,38^.

The bacterium *R. solanacearum* was cultured on casamino peptone agar (CPG) supplemented with 1% triphenyltetrazolium chloride (TZC) at 28 °C for two days. The bacteria were resuspended in sterile water at 10^9^ CFU mL^−1^. Bacterial suspensions (~50 mL) were applied to each plastic pot of tomato plants using the root inoculation method^39^. CFU were measured to calculate the bacterial populations using stem slices at 12 dpi^38^.

### Drought treatments and ion leakage assays

For drought treatments, a water-withholding experiment was performed for ~14 days, while control plants were maintained under well-watered conditions by regular watering. For ion leakage measurements, initial electrical conductivity was measured using a conductometer (SI Analytics, Mainz, Germany) after immersing ~0.2 g of tomato leaves in 20 mL of deionized water and shaking them at 28 °C for 2 h. Total conductivity was measured after the leaves were boiled at 95 °C for half an hour and returned to 25 °C. The electrolyte leakage is presented as the percentage of the initial conductivity of the total conductivity.

### Gene expression analysis

RNA was isolated using an RNA extraction kit (Easy-Do Biotech, Zhejiang, China) and reverse transcribed using a ReverTra Ace qPCR RT Kit (Toyobo, Tokyo, Japan) according to the manufacturers’ instructions. Quantitative real-time PCR (qRT-PCR) was conducted using a Light Cycler 480 II real-time PCR system (Roche, Basel, Switzerland). Each reaction buffer (20 µl) consisted of 10 µl of SYBR Green PCR Master Mix (Takara), 7.2 µl of water, 0.4 µl each of forward and reverse primers, and 2 µl of cDNA. The PCR conditions were as follows: 3 min at 94 °C, followed by 40 cycles of 30 s at 95 °C, 30 s at 58 °C and 1 min at 72 °C. The specific primers used for the target genes and internal control actin genes are listed in Supplementary Table S1.

### Subcellular localization

SlNAP1 was cloned into a pCAMBIA2300 (CAMBIA) vector with a GFP tag at the C-terminus under the control of the 35S CaMV promoter. Transgenic tobacco leaves were infiltrated with the resulting 35 S:SlNAP1-GFP construct mediated by *Agrobacterium tumefaciens* strain GV3101, according to the methods of Liao et al.^40^. The transgenic tobacco used in this experiment contained a nuclear localization protein that could emit red fluorescent signal (NLS-mCherry)^41^. At 48 h after infiltration, the fluorescence of the leaves was observed and recorded with a Zeiss LSM 780 confocal microscope; the excitation/emission wavelengths for GFP were 488 nm/500-530 nm and 561 nm/580–620 nm for NLS-mCherry. The primers used in this experiment are presented in Supplementary Table S1.

### Electrophoretic mobility shift assays and chromatin immunoprecipitation

To generate pET-32a-His-SlNAP1 constructs, the full-length CDS of SlNAP1 was cloned via PCR using specific primers (Supplementary Table S1). The PCR product was then inserted into a pET-32a vector between the Sac I and Hind III sites. The recombinant SlNAP1-His protein was expressed in *Escherichia coli* BL 21 (DE3) and purified following the instructions provided by the Novagen pET purification system. The ends of the oligonucleotide probes were labeled with biotin according to the instructions from a Biotin 3′ End DNA Labeling Kit (89818, Pierce) and annealed to double-stranded probe DNA. An electrophoretic mobility shift assay (EMSA) was conducted according to the instructions of a Light Shift Chemiluminescent EMSA Kit (20148, Thermo Fisher Scientific). The DNA probes used in the EMSA are shown in Supplementary Table S1.

Chromatin immunoprecipitation (ChIP) experiments were conducted with lateral leaflets from the upper leaves of mature 35S:SlNAP1-HA and WT plants following the instructions of a EpiQuik Plant ChIP Kit (P-2003-2, Epigentek). Approximately 1.5 g of leaf tissue was used and immunoprecipitated with anti-HA antibodies (26183, Pierce), with goat anti-mouse IgG antibodies (Millipore, AP124P) used as negative controls in this experiment. The primers used for ChIP-qPCR are listed in Supplementary Table S1.

### Measurement of phytohormones

Endogenous gibberellins (GAs) levels in tomato leaves were measured using a derivation approach coupled with nano-LC-electrospray ionization-quadrupole-time-of-flight-MS analysis^42,43^. SA and ABA in the tomato leaves were extracted and analyzed by light chromatography (LC)/tandem mass spectrometry (MS/MS) on a 1290 Infinity HPLC system coupled to a 6460 Triple Quad LC-MS device (Agilent Technologies), according to the methods of Wang et al.^44^.

### RNA-seq library preparation and sequencing

To extract RNA for sequencing, tomato leaves were collected at 12 h after *Pst* DC3000 infection and then immediately frozen in liquid nitrogen. Each treatment consisted of three biological replicates, and the samples of each biological replicate were taken from at least three plants. RNA-seq was conducted by staff at LC-Bio Technologies (Hangzhou, China).

Another RNA-seq dataset (6 h post-inoculation with *Pst* DC3000) was obtained from the “Tomato functional genomics database” (TFGD), and the transcriptome of tomato leaves treated with different bacteria and PAMPs was sequenced by BTI at Cornell University.

### Statistical analysis

At least three independent biological replicates sampled from different plants were included in each experiment. The data obtained were subjected to analysis of variance by SAS software, version 8 (SAS Institute), and the averages were compared using Tukey’s test (*P* < 0.05).

### Accession numbers

Sequence data from this article can be found in the Sol Genomics Network (http://solgenomics.net/) database under the following accession numbers: Solyc02g069960, Solyc03g083880, Solyc03g115850, Solyc04g009440, *SlNAP1* (Solyc05g007770), Solyc05g021090, Solyc05g055470, Solyc06g069710, Solyc06g074170, Solyc06g083850, Solyc07g063410, *SlACTIN* (Solyc03g078400), *SlGA2ox3* (Solyc01g079200), *SlPAL3* (Solyc09g007920), and *SlNCED1* (Solyc07g056570).

## Supplementary information

Supplementary information
