# Supplementary material for: Transcriptomic and genetic approaches reveal an essential role of the NAC transcription factor SlNAP1 in the growth and defense response of tomato
Source: Hortic Res. 2020 Dec 25;7:209. doi: 10.1038/s41438-020-00442-6 (PMC7759572; doi:10.1038/s41438-020-00442-6)
Supplement: Supplementary file 1 — Supplementary information [file 41438_2020_442_MOESM1_ESM.pdf]

Supplementary information

Transcriptomic and genetic approaches reveal an essential role of NAC transcription factor SlNAP1 in growth and defense of tomato

Jiao Wang, Chenfei Zheng, Xiangqi Shao, Zhangjian Hu, Jianxin Li, Ping Wang, Anran Wang, Jingquan Yu, Kai Shi

Fig. S1. Effects of *SlNAP1* overexpression on the net photosynthetic rate (Pn) in tomato leaves.

Table S1. Primers used in this study.

Table S2. 101 NAC transcription factors family and their gene expression ratio (*Pst* DC3000/Mock) after 6 and 12 h of *Pst* DC3000 infection in tomato.

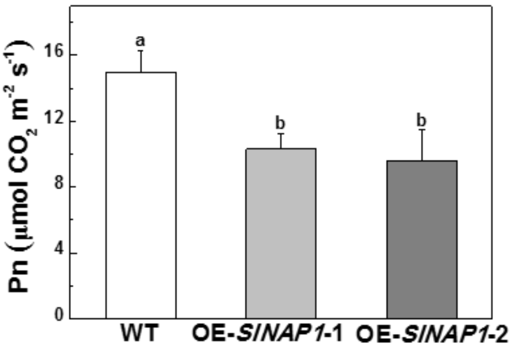

Fig. S1 Effects of *SlNAP1* overexpression on the net photosynthetic rate (Pn) in tomato leaves. The data were collected at 40 days after sowing. The results represent the average ± SD, n = 5. Different letters were used to indicate significant differences between genotypes ($P < 0.05$, Tukey's test).

Table S1 Primers used in this study.

Gene	Accession No.	Primer pairs	Assay
	Solyc02g069960	F: 5'-GCCAACAAAGCTCAGGAACA-3'<br>R: 5'-GTTCATCCCAGTTGCCATGT-3'	qPCR
	Solyc03g083880	F: 5’-TCCCATCTCATCACCACCAC-3'<br>R: 5’-TCCTCCTCTTGGCTGAGTTG-3'	qPCR
	Solyc03g115850	F: 5’-TTGAGGACAAACAGGGCAAC-3'<br>R: 5’-TTTCACCTCTTGGAGCCCTT-3'	qPCR
	Solyc04g009440	F: 5’-TTCAGATTCCATCCGACAGA-3’<br>R: 5’-CACCGTACAAAGCCTTCTCA-3’	qPCR
SlNAP1	Solyc05g007770	F: 5’-CCAAATAGAGCAGCTGTGTCA-3'<br>R: 5’-TTGGTGGTTTGCCTTTGTAA-3'	qPCR
		F: 5’-CgagctcATGGTTGGAAAAATTAGCTC-3'<br>R: 5’-TCCcccgggtCTGAGATTGAAATGTTGGATTC-3'	GFP fluorescence
		F: 5’-TTggcgcgccATGGTTGGAAAAATTAGCTC-3'<br>R: 5’-CGGggtaccCTGAGATTGAAATGTTGG-3'	Overexpression
		F: 5’-CgagctcATGGTTGGAAAAATTAGCTC-3'<br>R: 5’-CCCaagcttCTGAGATTGAAATGTTGG-3'	Prokaryotic expression
	Solyc05g021090	F: 5’-AGCAACTGGTATTGACAAGCC-3'<br>R: 5’-GTGGGAGACGAAACTCATGC-3'	qPCR
	Solyc05g055470	F: 5’-GCCGAGGTAAATGACCAACC-3'<br>R: 5’-AATGCAGGAGGAGCAGGAAT-3'	qPCR
	Solyc06g069710	F: 5’-TGAACCTTGGGACCTTCCAT-3'<br>R: 5’-CCAATACCCTGCAGCAGTTG-3'	qPCR
	Solyc06g074170	F: 5’-TTCCATGATCCCGGATAGGC-3'<br>R: 5’-GCCCTGGAAAGGAACAACAG-3'	qPCR
	Solyc06g083850	F: 5’-TCTTGAAACAACTCGATTGACC-3'<br>R: 5’-TCGAATCTCATGCCTTCGAG-3'	qPCR
	Solyc07g063410	F: 5’-TGGGTTCTTCCAAGTAAGGC-3’<br>R: 5’-CAGTTCCAGTTGCTTTCCAA-3’	qPCR
SlACTIN	Solyc03g078400	F: 5’-TGTCCCTATTTACGAGGGTTATGC-3'<br>R: 5’-CAGTTAAATCACGACCAGCAAGAT-3'	qPCR

SlGA2ox3	Solyc01g079200	F: 5’-ATGAGTTCTTCGTTTTCGTAGGG-3'<br>R: 5’-TTGTTGATTTGAGGAGGAGCA-3'	qPCR
		F: 5’-AGTTTGTCAAAGGTCGTGGCAACACCCCCACGCTTCTCTG-3'<br>R: 5’-CAGAGAAGCGTGGGGGTGTTGCCACGACCTTTGACAAACT-3'	EMSA
		F: 5’-AGTTTGTCAAAGGTatTGGCAACACCCCCAaGCTTCTCTG-3'<br>R: 5’-CAGAGAAGCtTGGGGGTGTTGCCAatACCTTTGACAAACT-3'	EMSA(mutant)
		F: 5’-CAAGTTTGTCAAAGGTCGTG-3'<br>R: 5’-GAGGGAATTAGAAGTTAGGAGT-3'	ChIP-qPCR
SlPAL3	Solyc09g007920	F: 5’-AGCTCCATGAATGGATCCTC-3'<br>R: 5’-CGATCAATGGATTGTCGTTC-3'	qPCR
		F: 5’-ATCACGATAAAAATAAATTAAGAGACACGAAAGTAAGACA-3'<br>R: 5’-TGTCTTACTTTCGTGTCTCTTAATTTATTTTTATCGTGAT-3'	EMSA
		F: 5’-ATCAaGATAAAAATAAATTAAGAGACAaGAAAGTAAGACA-3'<br>R: 5’-TGTCTTACTTTCtTGTCTCTTAATTTATTTTTATCtTGAT-3'	EMSA(mutant)
		F: 5’-TTAAAGCGTATGTATGTC-3'<br>R: 5’-TATCGGCTATCAGTTTGT-3'	ChIP-qPCR
SlNCED1	Solyc07g056570	F: 5’-GTTCGAAACGGAGCTAACCC-3’<br>R: 5’-TTGAACGGCGTGAACCATAC-3’	qPCR
		F: 5’-AGAAGAAAATAAAAACACACGTTTACAACATAAGATGATA-3'<br>R: 5’-TATCATCTTATGTTGTAAACGTGTGTTTTTATTTTCTTCT-3'	EMSA
		F: 5’-AGAAGAAAATAAAAACACAaGTTTACAACATAAGATGATA-3'<br>R: 5’-TATCATCTTATGTTGTAAACtTGTGTTTTTATTTTCTTCT-3'	EMSA(mutant)
		F: 5’-AAACGCTAAACTCAAAAG-3'<br>R: 5’-CAATGTGAACTAGAGGGA-3'	ChIP-qPCR

Table S2 101 NAC transcription factors family and their genes expression ratio (*Pst* DC3000/Mock) after 6 and 12 h of *Pst* DC3000 infection in tomato.

**Gene ID**	***Pst* DC3000 / Mock 6 h**		***Pst* DC3000 / Mock 12 h**	
	**ratio**	**adjust p**	**ratio**	**adjust p**
Solyc00g255510.1.1	1	NA	1	NA
Solyc01g009860.2.1	0.84	0.855172209	0.695660159	0.033694794
Solyc01g021730.2.1	1.87	1	1.017669955	1.00E+00
Solyc01g094490.2.1	1.58	0.445762789	0.960306335	0.868940423
Solyc01g102740.2.1	0.7	0.262667617	0.668724841	1.06E-01
Solyc01g104900.2.1	1.2	1	2.460365372	0.079989347
Solyc02g036430.1.1	1.54	1	1.338443442	0.367863323
Solyc02g061780.2.1	0.8	0.93874334	0.195597749	5.23E-07
Solyc02g061870.1.1	1	NA	0.49121292	0.038892862
Solyc02g061900.1.1	0.16	1	656.4730456	0.834658396
Solyc02g061910.1.1	0.15	0.743487341	1.001638584	1
Solyc02g062060.1.1	1	NA	0.25722447	7.66E-01
Solyc02g062070.1.1	1	NA	0.899920747	0.478790138
Solyc02g062090.1.1	1	NA	0.000792398	0.58686459
Solyc02g062210.1.1	8.33	1	0.84144471	0.28233441
Solyc02g069960.2.1	3.71	0.032462701	1.690476054	0.262128244
Solyc02g070140.1.1	1	NA	0.740669019	0.157043856
Solyc02g077610.2.1	1.4	0.926822475	1.301650319	0.511277393
Solyc02g081270.2.1	0.69	0.660068805	0.573053514	0.192689776
Solyc02g084350.2.1	1.28	1	2.430698306	1.97E-02
Solyc02g087920.2.1	8	0.944863217	1	NA
Solyc02g088180.2.1	1.14	1	1.790319664	2.35E-02
Solyc02g093420.2.1	1.47	0.662419829	0.664233388	1.14E-01
Solyc03g059300.1.1	1	NA	0.885863614	8.73E-01
Solyc03g062670.1.1	1	NA	0.98974235	1
Solyc03g062750.1.1	1	NA	0.28165014	0.268569905
Solyc03g078120.2.1	1.5	0.58559314	1.012865102	0.947462602
Solyc03g080090.2.1	0.88	0.815884485	0.846017268	0.247615307
Solyc03g083880.2.1	3.61	0.27302619	2.353233948	0.02699722
Solyc03g097650.2.1	0.67	0.637371143	1.868095121	0.000290973
Solyc03g098190.2.1	1.01	1	1.380386223	0.284990803
Solyc03g114220.1.1	1	NA	1	NA
Solyc03g114260.1.1	1	NA	1	NA
Solyc03g115850.2.1	2.18	0.82224825	2.768347937	0.000700898
Solyc04g005610.2.1	1.24	1	1.000032301	0.991770929
Solyc04g009440.2.1	6.22	1.73E-17	4.048179408	5.24E-19
Solyc04g015960.2.1	1.45	0.658453116	2.257358222	0.001366864
Solyc04g025760.1.1	1	NA	1.213790307	0.624167599

Solyc04g072220.2.1	1.15	0.979870793	1.12810686	0.42786854
Solyc04g078670.2.1	1.34	0.91683109	0.50482763	0.11388014
Solyc04g079940.2.1	1.48	0.339582105	1.067854138	0.703679972
Solyc05g007550.1.1	1	NA	1	NA
Solyc05g007770.2.1	2	0.648057501	11.34883394	9.64E-13
Solyc05g009840.2.1	1.55	0.227206801	1.324380417	0.061885914
Solyc05g010740.1.1	1	NA	1	NA
Solyc05g021090.2.1	4.22	0.001041201	2.147907228	0.027228799
Solyc05g026180.1.1	1.49	0.7185156	0.971358265	0.947461371
Solyc05g055470.2.1	1.55	0.18493815	1.788310529	8.90E-05
Solyc05g055480.2.1	0.97	1	0.830682553	0.218466505
Solyc06g008360.2.1	1.46	0.716691598	0.981994294	1
Solyc06g034340.1.1	1.18	1	1.019488534	1
Solyc06g060230.2.1	2.14	0.000638511	1.063522074	0.677900038
Solyc06g061080.2.1	0.36	0.26504361	0.736427749	6.34E-01
Solyc06g063380.1.1	1	NA	1	NA
Solyc06g063430.1.1	1	NA	1	NA
Solyc06g065410.2.1	1.83	0.93008205	1.04841793	1
Solyc06g068580.1.1	1	NA	1	NA
Solyc06g069100.1.1	1	NA	1	NA
Solyc06g069710.2.1	3.14	0.379523458	9.305672143	0.000908588
Solyc06g073050.2.1	1.26	0.769634919	1.239377339	0.396663248
Solyc06g074170.2.1	1.51	0.994580071	1.98000742	2.86E-02
Solyc06g083840.2.1	0.54	0.981886732	0.77650765	1.00E+00
Solyc06g083850.2.1	2.05	0.658453116	2.349182681	0.002957702
Solyc07g006840.1.1	1	NA	1	NA
Solyc07g045030.2.1	2.01	0.223760497	1.143502548	0.753232839
Solyc07g053590.2.1	1.62	0.948466591	1.281506773	0.511106107
Solyc07g053680.1.1	14.67	1	1	NA
Solyc07g062240.1.1	1	NA	1	NA
Solyc07g062840.2.1	0.69	1	1.185289924	0.816134146
Solyc07g063410.2.1	18.31	6.89E-38	18.45354656	6.49E-16
Solyc07g063420.2.1	0.45	0.487632726	0.233562196	0.000680081
Solyc07g066330.2.1	0.5	0.972895921	0.027171413	0.04329258
Solyc08g006020.2.1	0.95	1	0.862228003	7.79E-01
Solyc08g007020.1.1	1	NA	328.2365228	0.994761596
Solyc08g008660.2.1	5.33	1	3715.360123	0.116056012
Solyc08g028850.1.1	1	NA	1	NA
Solyc08g068380.2.1	0.71	0.58647546	1.285172067	0.448951353
Solyc08g074300.2.1	1	NA	8.367151996	1.44E-01
Solyc08g077110.2.1	0.98	1	1.894702356	0.29926912
Solyc08g079120.1.1	3	0.780734797	0.555115878	6.03E-01
Solyc09g010160.1.1	0.5	0.039989666	0.361868792	0.0000632
Solyc09g025310.2.1	3.86	0.001689545	0.470724485	6.99E-02

Solyc10g005010.2.1	0.72	1	0.90159586	1
Solyc10g006880.2.1	1	NA	1	NA
Solyc10g047060.1.1	19	0.850381896	3.98957033	0.731295037
Solyc10g055760.1.1	0.89	0.873774646	0.4886915	0.0000374
Solyc10g079220.2.1	1	NA	1	NA
Solyc10g083450.1.1	1	NA	0.000274121	0.318138951
Solyc11g005920.1.1	1.2	0.865266971	1.172678361	2.80E-01
Solyc11g008000.1.1	1.51	0.291544088	1.41653366	0.016081106
Solyc11g008010.1.1	1.36	0.520951574	1.426080507	0.069611877
Solyc11g017470.1.1	2.01	0.02065132	1.334191905	4.56E-02
Solyc11g018660.1.1	1.89	0.825843479	1.247748651	0.900287976
Solyc11g022480.1.1	1	NA	1	NA
Solyc11g065540.1.1	1	NA	1	NA
Solyc11g068620.1.1	0.72	0.837948036	0.341703618	0.05956828
Solyc11g068750.1.1	0.98	1	0.877259064	0.537914818
Solyc12g013620.1.1	1.38	0.711284962	2.131689978	8.67E-02
Solyc12g017400.1.1	1	NA	1	NA
Solyc12g036480.1.1	1	NA	1	NA
Solyc12g056790.1.1	1.35	0.591794621	1.231463678	0.247763224

Red: expression ratio >1.5, and RPKM>0

Blue: expression ratio >1.5, and RPKM=0
